# Development of passive CLARITY and immunofluorescent labelling of multiple proteins in human cerebellum: understanding mechanisms of neurodegeneration in mitochondrial disease

**DOI:** 10.1038/srep26013

**Published:** 2016-05-16

**Authors:** Jonathan Phillips, Alex Laude, Robert Lightowlers, Chris M. Morris, Doug M. Turnbull, Nichola Z. Lax

**Affiliations:** 1 Wellcome Trust Centre for Mitochondrial Research, Institute of Neuroscience, Newcastle University, Newcastle upon Tyne, NE2 4HH; 2 Bioimaging Facility, Newcastle University, Newcastle upon Tyne, NE2 4HH, United Kingdom; 3 Institute for Cell and Molecular Biosciences Newcastle University, Newcastle upon Tyne, NE2 4HH, United Kingdom; 4 Medical Toxicology Centre, Newcastle University, Wolfson Building, Claremont Place, Newcastle NE2 4AA.

## Abstract

CLARITY enables immunofluorescent labelling and imaging of large volumes of tissue to provide a better insight into the three dimensional relationship between cellular morphology and spatial interactions between different cell types. In the current study, we optimise passive CLARITY and immunofluorescent labelling of neurons and mitochondrial proteins in mouse and human brain tissues to gain further insights into mechanisms of neurodegeneration occurring in mitochondrial disease. This is the first study to utilise human cerebellum fixed in paraformaldehyde and cryoprotected in conjunction with formalin-fixed tissues opening up further avenues for use of archived tissue. We optimised hydrogel-embedding and passive clearance of lipids from both mouse (*n* = 5) and human (*n* = 9) cerebellum as well as developing an immunofluorescent protocol that consistently labels different neuronal domains as well as blood vessels. In addition to visualising large structures, we were able to visualise mitochondrial proteins in passively cleared tissues to reveal respiratory chain deficiency associated with mitochondrial disease. We also demonstrate multiple use of tissues by stripping antibodies and re-probing the cerebellum. This technique allows interrogation of large volumes intact brain samples for better understanding of the complex pathological changes taking place in mitochondrial disease.

One of the major goals of neurodegenerative research is to further understand the pathological processes underpinning neurological disease and to drive the development of treatment in the earliest stages of disease. Neurodegenerative research relies on the availability of human post mortem brain samples to characterise the neuropathological features using light-based imaging approaches including histochemical and molecular labelling methods. These approaches tend to focus on thin (5–20 μm) sections of brain tissue and in these sections, it can be difficult to track individual neurons and their connections in a three dimensional (3D) volume using serial tissue sections. Recently, high resolution techniques, such as array tomography and serial block-face scanning electron microscopy, have been developed although these can be time consuming, technically difficult, expensive and restricted to small tissue volumes^1^,^2^. A major limitation associated with light based microscopy analysis of thicker sections stems from the fact that the CNS is heterogeneous giving rise to different refractive indices across the tissue, and also a high lipid content which can cause light scattering, restricting the ability to image thicker sections. Multi-photon imaging overcomes this to a degree since it allows imaging of sections across hundreds of microns however it is not possible to image the entire brain.

A number of methods have focussed on addressing the problem of investigating thicker tissue sections with the aim of minimising the light scattering properties of tissue by either removing the lipids from the tissue, or altering the refractive indices of tissue to make it more uniform, or a combination of the two. Clear Lipid-exchanged Acrylamide-hybridized Rigid Imaging compatible Tissue hYdrogel (*CLARITY*) is a new technique which allows interrogation of large volumes of tissues to reconstruct the 3D architecture of the brain and provide insights into abnormalities associated with neurodegeneration disease^3^. *CLARITY* works by allowing covalent binding of hydrogel monomers to primary amine groups of molecules within the tissue to form a hydrogel matrix, once formed the tissue is then subjected to lipid clearing methods while the structure remains intact and proteins, DNA and RNA are retained. The benefit of incorporating the native biological molecules into the hydrogel matrix is that there is negligible protein loss following the clearing step^3^,^4^. Once the clearing step has been completed, the tissue sections are then incubated in an imaging solution to further change the refractive index of the tissue and reduce light scattering. The technique produces transparent tissue on a large-scale and as the hydrogel is porous, it allows the diffusion of antibodies during immunostaining protocols on both mouse and human tissue.

In light of recent papers utilising CLARITY to investigate neuronal changes in human tissue with neurological disorders such as Alzheimer’s disease^5^, Parkinson’s disease^6^ and autism^3^, we aimed to optimise the technique to enable the investigation of pathological changes which frequently occur in the cerebellum of patients with mitochondrial disease. Cerebellar ataxia is commonly reported in mitochondrial disease and neuropathological findings report microinfarcts, Purkinje cell loss, axonal torpedoes and mitochondrial respiratory chain defects^7^,^8^. The cerebellum has a well-defined circuitry receiving glutamatergic innervation from the climbing fibres and mossy fibres which synapse on Purkinje cells. The Purkinje cells are sandwiched between the molecular and granular cell layers in the cerebellar cortex and project their GABAergic axons into the deep cerebellar nuclei. There have been a number of studies documenting Purkinje cell abnormalities in mitochondrial disease. Therefore in this study we adopt an integrative approach to understand the impact of mitochondrial defects on the 3D cerebellar circuitry using CLARITY. We report the development of an improved passive CLARITY technique, quadruple immunofluorescent staining using multiple markers and confocal microscopy imaging of human post-mortem cerebellum.

## Results

We first report the optimal methods for passively clearing 4% paraformaldehyde (PFA)-fixed mouse cerebellum before applying this to formalin-fixed human cerebellum tissues since these are a limited and valuable resource. We describe the ideal conditions to perform immunofluorescent labelling of neurons, their connections and mitochondrial proteins using various antibodies to further understanding of cerebellum connectivity in normal and pathological conditions.

### Mouse Cerebellum

To optimise hydrogel embedding of brain tissue, we used pre-sectioned and whole cerebellum from five wild type 12 month old C57/Bl6 mice. Following 3 days of hydrogel incubation at 4 °C, samples were transferred to a 37 °C water bath to initiate polymerisation. After 4 hours, the hydrogel polymerised forming a strong hydrogel matrix around the tissue. Excision of the tissue from excess hydrogel matrix was straightforward for the whole cerebellum while the pre-sectioned cerebellum was easily damaged. Therefore, for further processing only whole cerebellum samples were embedded in hydrogel, polymerised and then sectioned at various section thickness (250–500 μm) using a vibratome.

**Figure 1:**
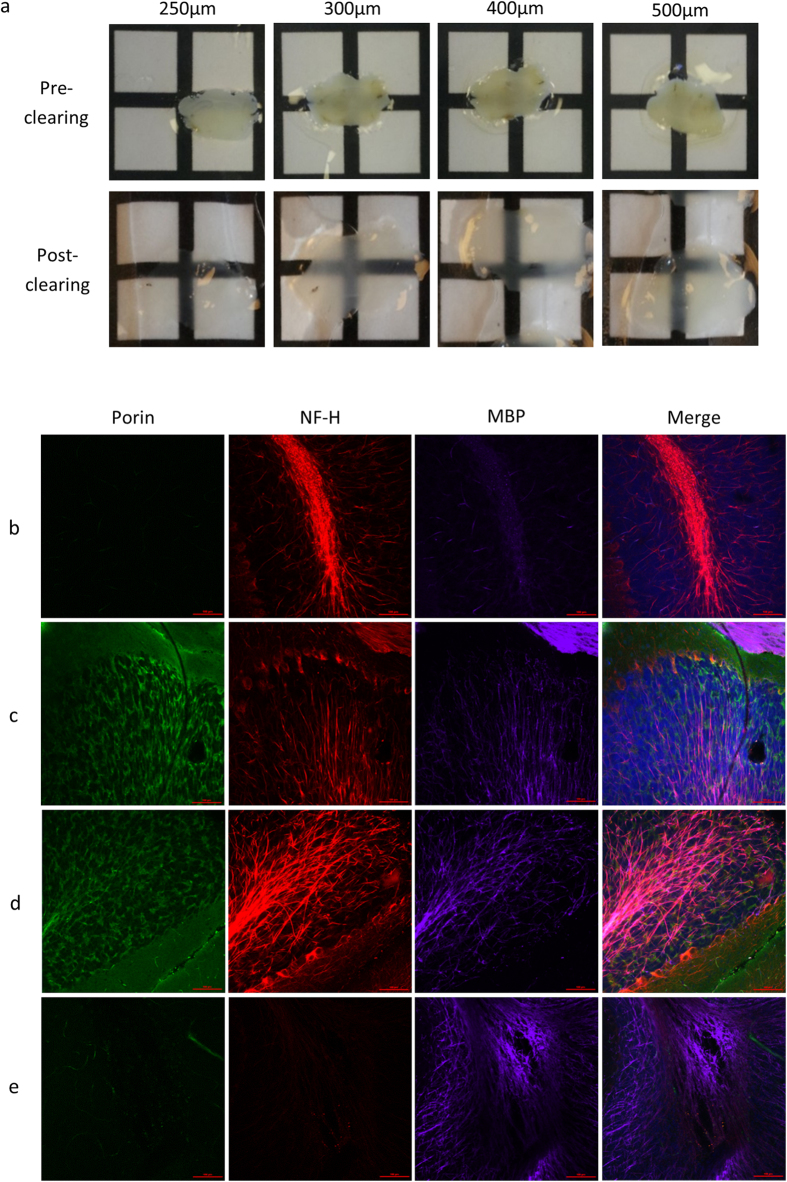
Demonstration of passive CLARITY and optimisation of immunofluorescent labelling conditions on wild type mouse brain sections. Representative images of wild type 12 month old C57/Bl6 mouse cerebellar sections of varying thickness are shown pre- and post-passive clearance (**a**). Passively cleared cerebellar sections were immunofluorescently labelled with antibodies raised against porin (green), neurofilament H (NF-H; red) and myelin basic protein (MBP; purple). Various conditions were tested for the immunolabelling protocol; (**b**) sodium borate buffer at 37 °C for 24 hours, (**c**) sodium borate buffer at 4 °C for 6 days for the primary antibodies, then at 4 °C for 4 days for secondary antibodies, (**d**) PBST at 4 °C for 6 days for the primary antibodies, then at 4 °C for 4 days for secondary antibodies and (e) The advantages of passively clearing tissue sections is exemplified in an uncleared section which was stained using the protocol in (**d**). Scale: 100 μm.

A number of recent studies have identified issues with electrophoretic-based active clearance techniques and resultant tissue damage^9^,^10^. Given this and the recent success of passive clearance techniques^4^,^11^, we chose to use a passive clearing approach. Mouse cerebellum of variable section thickness was rendered transparent by incubation in clearing buffer at 37 °C for 7 days (Fig. 1a). There was a noticeable increase in tissue expansion following passive incubation in the clearing solution which is visible in Fig. 1a. This has been previously reported by others using both passive and active clearing processes and is resolved once samples are immersed in mounting solution prior to imaging without negative effects on the cellular morphology or protein content^3^,^4^.

### Optimisation of immunofluorescent protocol on passively cleared 250 μm mouse cerebellum sections

The original study by Chung *et al*. utilised a staining protocol whereby primary and secondary antibodies were diluted in sodium borate (SB) buffer and incubated at 37 °C for 24 hours each, with 12 hour washes in between^3^. To test this method, we used an antibody to label neurons and their processes (anti-neurofilament H 200 kDa (NF-H)), myelin (anti-myelin basic protein (MBP)) and mitochondria (anti-porin) since these antibodies are consistently reliable in 5 μm sections (Supplementary Figure 1). Replicating Chung and colleagues approach, we found that using cleared sections of 250 μm thickness and incubating with antibodies diluted in SB buffer at 37 °C for 24 hours produced only weak staining for neurofilament H, and an almost total lack of staining was observed for myelin basic proteins and porin (Fig. 1b). To improve immunofluorescent labelling, we reduced the temperature to 4 °C since a lower temperature increases the specificity of antibody binding and increased the length of incubation to 6 days for the primary antibodies and 4 days for the secondary antibodies. In this trial, we compared the use of SB buffer and PBS with 0.1% tween-20 (PBST) as a diluent. Both SB buffer (Fig. 1c) and PBST (Fig. 1d) at the lower temperature with extended incubation periods produced specific staining of mitochondria, neurons and axons and myelin. However, the SB buffer formed crystals both in the solution and on the sections at this lower temperature. Therefore we used primary antibodies diluted in PBST and incubated with tissue at 4 °C for 6 days following by extensive washing and diluting secondary antibodies in PBST at 4 °C for 4 days for immunofluorescent staining on mouse sections. To confirm the necessity of clearing we stained an unclear section using the 4 °C prolonged incubation step (Fig. 1e) which revealed weak staining of myelin and absent labelling of mitochondria or neurons.

### Poor antibody penetration in passively cleared mouse cerebellar sections

Confocal imaging of immunofluorescently labelled sections at different thicknesses revealed issues with antibody penetration which was notably reduced above 300 μm (Supplementary Figure 2). The initial and final z stacks to 150 μm within the sections revealed strong and uniform staining that decreased dramatically towards the middle of the section. Poor antibody penetration has been observed previously in a study by Ando *et al*.^5^. To improve permeability of the tissue, we tested pre-treatment of the tissues in PBS-Triton X and proteinase K (10 mg/ml) solution for 10 minutes^12^, however proteinase K treatment caused the tissue to disintegrate and minimal improvement was seen with PBS-Triton X. Therefore the remainder of the study was conducted using 250 μm thick sections.

### Optimisation of reliable antibodies in mouse tissue

**Figure 2:**
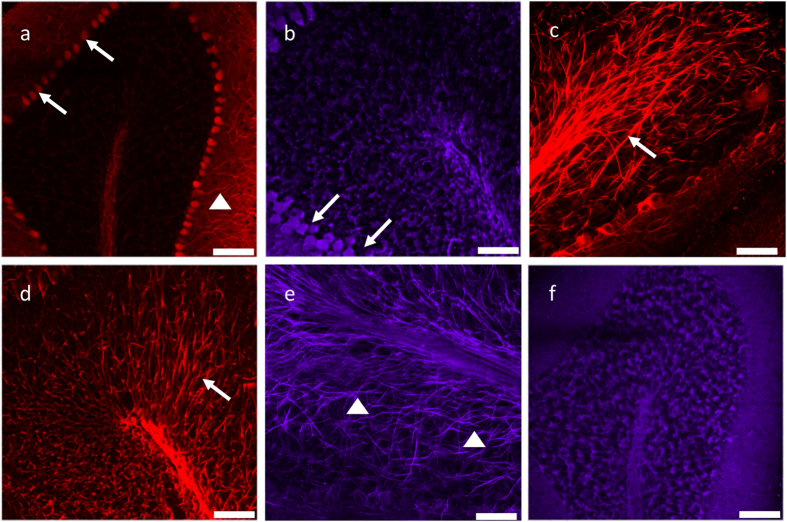
Representative images of immunofluorescent labelling of neurons, axons and mitochondrial proteins in passively cleared 250μm mouse cerebellum. Passively cleared 250 μm wild type mouse cerebellum sections were stained for both neuronal and mitochondrial proteins commonly found in the cerebellum. Calbindin ((**a**); red; 546 nm) and parvalbumin ((**b**); purple; 647 nm) antibodies positively label Purkinje cell bodies. Purkinje cell axons were visualised through positive labelling of neurofilaments ((**c**); red; 546 nm; NF-H, and (**d**); red; 546 nm; SMI-31) and myelin ((**e**); purple; 647 nm; MBP). Mitochondria were detected through positive labelling of COXI protein ((**f**); purple; 647 nm; COXI). Scale bar: 100 μm.

The outlined immunostaining method demonstrates labelling of neurons, myelin and mitochondria in the cerebellar cortex however we now wanted to confirm that it is possible to label other components of the cerebellum (Fig. 2). To specifically visualise Purkinje cell bodies, we labelled calbindin (anti-calbindin D-28k) and parvalbumin (anti-parvalbumin) since they are both calcium binding proteins expressed in cerebellar Purkinje cells. Both antibodies produced specific labelling of the Purkinje cell bodies (Fig. 2a,b, white arrows) with calbindin also labelling the dendrites of Purkinje cells (Fig. 2a, white arrow head). To document changes in Purkinje cell connectivity, Purkinje cell axons were labelled with NF-H (Fig. 2c, white arrow) and phosphorylated neurofilaments (Fig. 2d, white arrows) to observe Purkinje cell axons projecting from the Purkinje cell layer through the granular cell layer to the deep white matter. The myelin sheath surrounding Purkinje cell axons in the granule cell layer can also be visualised through the labelling of MBP (Fig. 2e, white arrowhead). To visualise mitochondria, antibodies raised against an outer membrane protein, porin (anti-porin) and a subunit of the mitochondrial respiratory chain complexes, complex IV subunit I (anti-COXI) were used and showed positive immunoreactivity (Fig. 2f). All optimal antibody conditions are given in Table 1.

### Human Cerebellar sections

**Figure 3:**
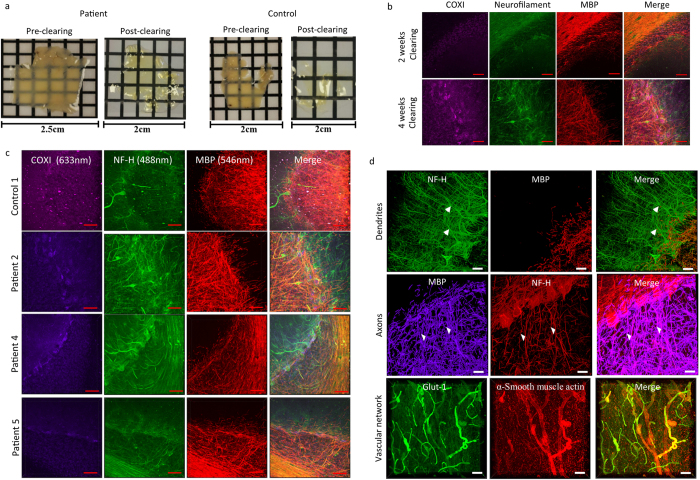
Demonstration of passive CLARITY and immunofluorescent labelling of different neuronal subcompartments, blood vessels and mitochondria. 250 μm human cerebellar sections from control individuals and a patient with mitochondrial disease underwent passive clearing at 37 °C for 4 weeks (**a**). The quality of immunofluorescent staining is determined by duration of passive clearing; 2 weeks of passive clearing produced minimal labelling of the white matter in the granule cell layer (NF-H; green; 488 nm and MBP; red, 546 nm) with an absence of labelling of mitochondria (COXI; purple; 647 nm; (**b**)). Extending passive clearing to 4 weeks improved the quality of stain with identifiable Purkinje cells and their axons (NF-H, green; 488 nm) and their myelin sheaths (MBP; red, 546 nm) and mitochondria (COXI; purple; 647 nm; (**b**)). In cerebellum subjected to passive clearing for four weeks, positive labelling of mitochondria (COXI; purple; 647 nm) and myelinated Purkinje cell axons (NF-H; green; 488 nm and MBP; red; 546 nm) were identified post-mortem human cerebellum tissue from controls and patients with mitochondrial disease (**c**). Using a combination of antibodies, it is possible to label neurons and track their three dimensional projections, including Purkinje cell (NF-H), axons (NF-H and MBP) and their dendritic arborisations (NF-H; (**d**)). It is also possible to detect the vascular network (Glut-1 and α-smooth muscle actin; (**d**)). Scale: 100 μm.

We applied our optimised method on human cerebellum in order to visualise connections within control individuals (*n* = 2) and patients with clinically and genetically confirmed mitochondrial disease (*n* = 5). The human cerebellum sections required longer incubation in clearing solution in order to become transparent, with tissues taking at least four weeks to clear (Fig. 3a). The length of clearing is imperative for the quality of staining achieved, since sections that were only cleared for 2 weeks revealed weak staining of the three markers that were applied (anti-COXI, anti-NF-H and anti-MBP; Fig. 3b). Only a small proportion of the myelin sheath was labelled while a limited number of axons were also labelled. However when the length of passive clearing was extended to four weeks, there was extensive labelling of the myelin sheath in the granule cell layer, clear labelling of mitochondria in the Purkinje cell soma and a higher density of axons (Fig. 3b).

Anti-COXI, anti-NF-H and anti- MBP were applied to the passively cleared 250 μm human cerebellar sections using PBST as the diluent at 4 °C and were incubated for 6 days and 4 days for the primary and secondary antibodies, respectively. From the seven cases, there were mixed results. There was positive labelling of both NF-H and MBP allowing for the visualisation of myelinated Purkinje cell axons in the granule cell layer of control 1 and Patients 2, 4 and 5 (Fig. 3c). While in the case of control 2, patient 1 and 6 there was no specific staining of any antibody. We are unsure why staining of these tissues was not successful since the length of fixation and post-mortem interval (PMI) of control 2 (fixation: 8 years, PMI: 39 hours), patient 1 (fixation: 6 years, PMI: 21 hours) and 6 (fixation: 2 years, PMI: 76 hours) falls within the range of successful cases (the only exception being control 2 where fixation 1 year over this range) (fixation: 3–7 years, PMI: 21–112 hours). Control 2 demonstrated non-specific staining of the blood vessels and aggregation of the fluorophores in the 488 nm channel. There was some specific staining of both NF-H and MBP in Patient 3, however it was only the larger thicker axons that were labelled.

The human sections that have been used in this study were fixed in formalin for a maximum length of seven years while in other studies tissues were fixed for six years^3^,^5^,^6^. In addition to formalin-fixed sections, Liu and colleagues applied the *CLARITY* protocol to fresh brain tissue and found that fresh cortical sections took a shorter amount of time to passively clear (~40 days) compared to formalin-fixed cortical sections (~60 days). On the basis of the success in fresh tissue and the availability of human tissue that had been fixed in PFA then frozen at the NBTR, we applied the CLARITY protocol to human cerebellar sections that had been PFA-fixed then frozen rather than archival formalin-fixed sections. The PFA-fixed and frozen section (control 3) passively cleared in 6 days and there was positive staining of NF-H in the sections (Supplementary Figure 3) confirming that in addition to formalin-fixed sections, PFA-frozen sections are also a useful resource.

### Imaging of structures in the passively cleared human cerebellum sections

Since we were able to visualise Purkinje cells and mitochondria in human cerebellar sections, we performed immunofluorescent staining and confocal microscopy on passively cleared human sections (250 μm) to visualise a variety of different cellular domains including dendrites, axons and the vascular network (Fig. 3d). Anti-NF-H was used to label the Purkinje cells and their dendrites (Fig. 3d) while both anti-NF-H and anti-MBP, label myelinated axons (Fig. 3d). Confocal images were reconstructed into 3D stacks allowing for the visualisation of the intricate processes of both structures. To visualise the blood vessels, anti-α smooth muscle actin and anti-glut-1 were used as markers for smooth muscle cells and endothelium respectively (Fig. 3d). Through labelling glut-1, capillaries were observed throughout the 250 μm thick section and co-localisation of glut-1 and α smooth muscle actin was observed in arterioles. The 3D morphology was reconstructed using Imaris software (Supplementary video 1).

### Visualisation of mitochondria in passively cleared human cerebellum sections

Following the success of visualising large structures such as axons, dendrites and the vascular network in both control individuals and patients with mitochondrial disease, we then sought to determine if it was possible to visualise mitochondrial respiratory chain proteins in passively cleared cerebellar sections for the purpose of defining mitochondrial respiratory chain deficiency.

**Figure 4:**
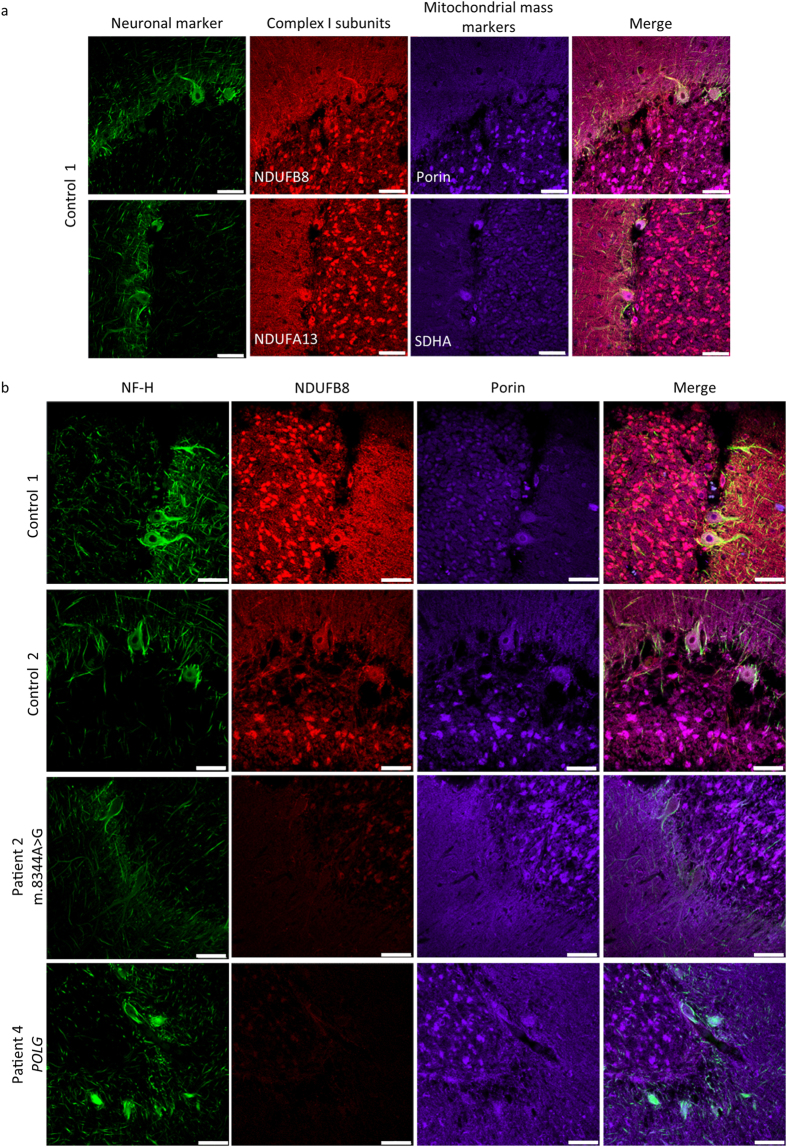
Assessment of mitochondrial respiratory chain deficiency in Purkinje cell neurons in human cerebellum. 250 μm-thick cerebellum section were passively cleared and immunofluorescently labelled to identify mitochondrial mass (porin and SDHA, 647 nm) and complex I subunits within the mitochondrial respiratory chain (NDUFB8 and NDUFA13; 546 nm) in conjunction with a neuronal marker (NF-H; 488 nm) in control 1 (**a**). To interrogate respiratory chain deficiency (reduced abundance of a complex I subunit relative to mitochondrial mass) in Purkinje cells and their projections in patients with mitochondrial disease neurons (NF-H; 488 nm), mitochondria (porin; 647 nm) and complex I subunit (NDUFB8; 546 nm) were immunofluorescently labelled. Control Purkinje cells reveal matched abundance of NDUFB8 protein relative to porin protein (control 1 and 2), while patient Purkinje cells and surrounding neurons showed an absence of NDUFB8 protein relative to porin protein level confirming the presence of complex I deficient mitochondria in these cells (patient 2 and 4; (**b**)). Scale: 100 μm.

Previous studies have used a mitochondrial mass marker in combination with a complex I antibody to determine if there is downregulation of complex I expression, a sign of respiratory chain deficiency in mitochondria^13^,^14^. In passively cleared control sections, complex I subunits NDUFB8 and NDUFA13 were tested in conjunction with the mitochondrial mass markers, porin and SDHA (for complex II). There was positive staining of all the mitochondrial antibodies with both NDUFB8 and NDUFA13 co-localising with their respective mitochondrial mass markers, thereby confirming positive and specific staining of complex I subunits (Fig. 4a). The neuronal marker NF-H was used as positive control to confirm that the immunolabelling protocol was successful.

After the successful labelling of mitochondrial proteins, we then compared respiratory chain deficiency in passively cleared cerebellar sections from patients with mitochondrial disease and control subjects. Cerebellar sections from control 1, control 2, patient 2 (m.8344 A > G) and patient 4 (*POLG*) were stained for NF-H (to serve as a neuronal marker), NDUFB8 (complex I marker) and Porin (mitochondrial mass marker; Fig. 4b). Only these sections were used for this optimisation step since they consistently produced the best results following each round of immunolabelling. In control sections there is co-localisation between NDUFB8 and porin confirming that there is no respiratory chain deficiency. In both patients there is a clear and global loss of NDUFB8 relative to porin which confirms respiratory chain deficiency in patients. The loss of NDUFB8 labelling compared to porin confirms that it is possible to visualise respiratory chain deficiency in mitochondria within multiple neuronal subcompartments within passively cleared sections and importantly allow documentation of the distribution of respiratory chain deficiency within individual neurons, their dendrites and axons.

### Quadruple immunofluorescent staining in passively cleared human cerebellum sections

**Figure 5:**
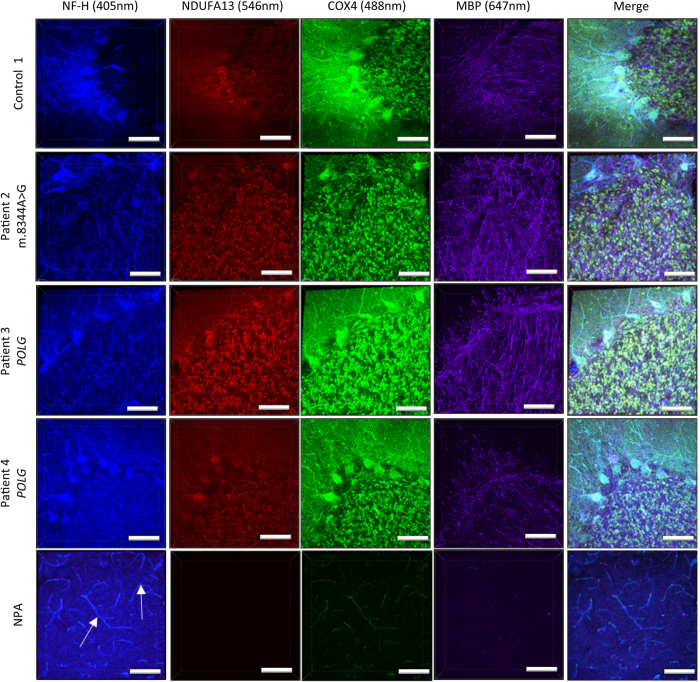
Representative images of Purkinje cells and their myelinated axons in human cerebellum following quadruple immunofluorescence. Passively cleared 250 μm thick human cerebellar sections were stained for neurofilament H (blue), NDUFA13 (red), COX4 (green) and Myelin basic protein (purple). Mitochondria are clearly visible through the positive labelling of NDUFA13 (red) and COX4 (green). The myelin sheaths (Myelin basic protein, 647) of the Purkinje cell axons have been clearly labelled. In the 405 nm channel there is a high level of autofluorescence making it difficult to visualise Purkinje cell axons (neurofilament H). The presence of autofluorescence in the 405 nm and 488 nm channel was confirmed through a no primary antibody control section. Scale: 100 μm.

Previous studies investigating mitochondrial respiratory chain abnormalities in post-mortem brain tissue have focused on immunofluorescent staining on thin sections^14^, which are limited by the amount of information that can be captured about neurons and their projections. Using 250 μm clarified patient cerebellum, we tested the feasibility of quadruple immunofluorescent assessment of mitochondrial respiratory chain complex I and mitochondrial mass in neurons and their associated myelinated axons. In order to achieve this we used two mitochondrial markers; a marker for a subunit of complex I (NDUFA13) and a marker for nuclear-encoded complex IV (COX4) for mitochondrial mass and then NF-H and MBP to identify neurons and their myelinated axons (Fig. 5). The secondary antibodies used were conjugated to different fluorophores including Alexa-405 nm (for NF-H), Alexa-488 nm (for COX4), Alexa-546 nm (for NDUFA13) and Alexa-647 nm (for MBP) to allow spectral distinction of the different proteins, and this was the first time that the 405 nm fluorophore had been used on a passively cleared piece of tissue. The results from the best cases are depicted in Fig. 5, with the specific staining of the intricate network of myelinated axons clearly visible; COX4 and NDUFA13, positively labelled complex I and IV in mitochondria in the granule cell layer and Purkinje cell soma showing good colocalisation in control 1. In patient tissues, both patient 2 (m.8344 A > G) and patient 3 (*POLG*) had a similar expression of NDUFA13 compared to COX4 while patient 4 (*POLG*), the expression of NDUFA13 in the Purkinje cell bodies appears reduced compared to COX4. These observations are in agreement with a previous study by Chrysostomou and colleagues that used immunofluorescence in thin sections to investigate respiratory chain deficiency^13^.

However, there is a high level of autofluorescence in the 405 nm channel, confirmed by no primary control, making it difficult to visualise NF-H staining. In these sections, there is a high level of background noise and blood vessels are clearly visible (white arrow) in the 405 nm channels.

### Reusing previously stained passively cleared human cerebellar sections

As the native proteins are incorporated into the hydrogel, a proposed advantage of the CLARITY method, is the ability to reuse stained cleared sections^3^. Therefore there would be a reduced risk of protein loss when using a detergent to strip away the old antibodies.

**Figure 6:**
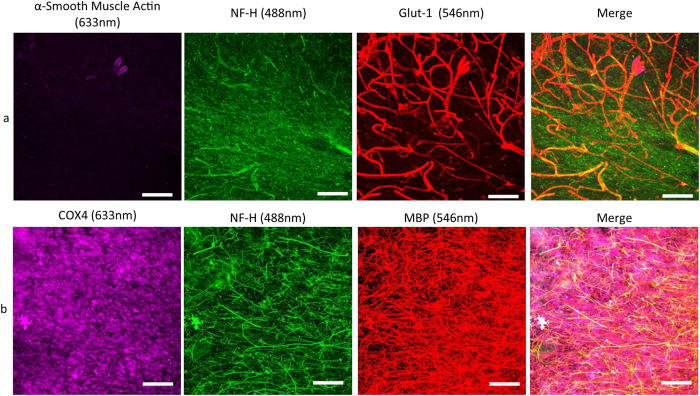
Reusing passively cleared and stained 250 μm thick control human cerebellum sections. Passively cleared sections were originally stained for α-smooth muscle actin (purple; 647 nm), neurofilament H (green; 488 nm) and Glut-1 (red; 546 nm) producing a clear image of the cerebellar vascular network (**a**). Following a week long incubation in the clearing solution, the antibodies were removed and the sections were then successfully stained with COX1 (purple; 647 nm), neurofilament H (green; 488 nm) and myelin basic protein (red; 546 nm) demonstrating the distribution of mitochondria in myelinated axons (**b**). Scale: 100 μm.

A section that had been previously stained and imaged with α-smooth muscle actin, NF-H and glut-1 antibodies (Fig. 6a) underwent a second step of clearing at 37 °C for one week. Once the clearing step was completed, the section was imaged to confirm that none of the previous staining was present. There was no labelling of α-smooth muscle actin, NF-H or glut-1 in the newly cleared section (Fig. 6b). The section was re-stained with COX1, NF-H and MBP. Imaging of this section show labelling of the axons and myelin sheath (Fig. 6c). No blood vessels were visible confirming that the previous staining had been completely removed and that there was no protein loss as there was successful staining in the second round.

All optimal conditions for primary and secondary antibodies used on passively cleared human tissues are given in Supplementary Table 1 and Supplementary Table 2, respectively.

## Discussion

In conclusion we have shown successful passive clearing of both fresh and fixed mouse sections as well as archival formalin-fixed human tissue sections from both control individuals and patients with mitochondrial disease. We have also demonstrated for the first time that PFA-fixed cryoprotected frozen human cerebellar sections are suitable samples for *CLARITY* which increases access to and analysis of archived material. Using passively cleared sections, we trialled a number of immunolabelling conditions starting with previously published methods^3^, and observed that our protocol of using PBST as a diluent with prolonged antibody incubations at 4 °C gave optimal staining. The optimal conditions for all antibodies used in this study are summarised in Table 1. Using these antibodies, we were able to visualise dendrites, axons, Purkinje cell bodies and the vasculature network within the cerebellum. In addition to those large structures, for the first time, we were able to image mitochondrial proteins in passively cleared cerebellum and were able to detect respiratory chain deficiency in patients with mitochondrial disease. The respiratory chain deficiency observed agrees with previous reports in the same individuals further validating this approach^13^. The ability to image mitochondrial proteins in passively cleared volumes of tissues will advance understanding of the distribution of mitochondrial respiratory chain deficiency in different compartments of the same neuron and provide both spatial and structural information about those affected cells. This will allow us to ask and answer questions about where respiratory chain deficiency develops first in the neuron, and how this compares to other neighbouring cells in the brain.

We have also made the first attempt to quadruple stain passively cleared sections, however our efforts are not without complication as there is a high level of autofluorescence in the 405 nm channel which cannot be circumvented by glycerine treatment. One of the major advantages of *CLARITY* is the ability to reuse multiple tissue volumes that have previously undergone immunofluorescent staining^3^. Replication of these findings has not been reported, however we confirmed that a seven day incubation in the clearing solution was sufficient to remove all antibodies and subsequent to the clearing process, positive labelling of different structures is made possible. This property of *CLARITY* is highly valuable since it allows for the reuse of a rare resource such as post-mortem human sections from individuals with a rare disorder such as mitochondrial disease, and could allow reconstruction of the same piece of tissue with different cellular or organelle markers.

The combination of *CLARITY* and our immunolabelling protocol will open new avenues into the neuropathological research of mitochondrial disease. *CLARITY* will enable exploration of the global changes to Purkinje cell connectivity and how mitochondrial dysfunction might contribute to an irreversible loss of Purkinje cells. Additionally we will be able to look at the complex vascular structure in both non-lesioned and lesioned regions of patients with mitochondrial disease and compare these with control individuals to help determine vasogenic factors in the formation of focal necrotic lesions which are common in patients with mitochondrial disease. This will not only benefit neuropathology of mitochondrial disease but also common neurodegenerative disorders, as already shown^5^,^6^, as it will allow for the investigation of global changes to vascular, dendritic or axonal networks in sections to provide insight to how the neuronal population reacts to a degenerative stimuli.

## Methods

### Mice

Five wild type 12 month old C57BL/6 mice were used in the study. Mice were culled using cervical dislocation. The brains were then excised and incubated in 4% PFA at 4 °C overnight and then washed in PBST. All animal experimental procedures were conducted in accordance with the guidelines of the UK Home Office guidelines and under its approval (licence 60/4455).

### Human tissue

Formalin-fixed cerebellar brain tissue was obtained from six patients (age range: 55–79 years) with genetically and clinically confirmed mitochondrial disease, and two control individual (age: 72 years and 77 years) with no clinical or pathological evidence of neurological disease (see Table 2). A PFA-fixed and frozen control section with no clinical or pathological evidence of neurological disease was also used in this study (Control 3). The tissue was obtained through the Newcastle Brain Tissue Resource (NBTR) and sections (~3 cm width) containing at least two cerebellar folia were removed from large fixed cerebellum sections. The sections were washed in PBST at 4 °C overnight. The study was approved by Newcastle and North Tyneside Local Research Ethics Committee. All human material were used in accordance with the Declaration of Helsinki. The Newcastle Brain Tissue Resource is covered by the Newcastle University Human Tissue Authority license as a research tissue bank. All tissue was collected with informed consent.

### Hydrogel Preparation

The hydrogel solution was prepared by combining 20 ml of acrylamide (40%, Bio-Rad Laboratories), 10 ml bis-acrylamide (2%, Bio-Rad Laboratories), 1 g VA-044 initiator (0.25% w/v, Alpha Laboratories), 40 ml 10 X PBS and 310 ml _d_H_2_O. This was completed on ice and then stored at −20 °C until needed.

### Tissue embedding

The tissue was placed in 50 ml of hydrogel solution and incubated for 3 days (mice) or 7 days (human) at 4 °C. Once the incubation was completed, the Falcon tubes were opened and placed in a desiccation chamber where it was filled with nitrogen for 10 minutes. A vacuum pump then removed all gas from the desiccation chamber for 10 minutes before being refilled with nitrogen from a tank for another 10 minutes. The Falcon tubes were resealed ensuring exposure to the atmosphere was minimal. The Falcon tubes were transferred to a 37 °C water bath for 3 hours to initiate the polymerisation of the hydrogel.

### Passive Tissue Clearing

Once the hydrogel had polymerised, the tissue was removed in a fume hood. The tissue was washed in PBS overnight and then sectioned to 250 μm on a vibratome. The clearing solution was prepared by combining 24.722 g boric acid (Sigma Aldrich), 80 g sodium dodecyl sulphate (Sigma Aldrich), and 2ltr _d_H_2_O and pH adjusted to 8.5 by adding NaOH. The 250 μm sections were placed in a 50 ml Falcon tube and filled with 40 ml clearing solution before being placed in a 37 °C water bath. The clearing solution was changed every other day. The length of time for passive clearing was assessed by eye but for mouse 250 μm cerebellar sections typically cleared in 7 days while for human 250 μm cerebellar sections it was >4 weeks.

### Immunofluorescent staining

Once clearing was comple, the sections were washed with PBS five times for 30 minutes and kept in PBS at 4 °C until required. The sections were then incubated in normal goat serum (10% v/v, Sigma Aldrich) for 90 minutes to block non-specific binding of secondary antibodies. The properties of the antibodies used in the study are summarised in Supplementary Table 2.

For labelling of mitochondrial proteins, the markers of respiratory chain deficiency (NDUFB8 and NDUFA13) were biotinylated. The immunolabelling protocol consisted of a 6 day incubation of primary antibodies (NDUFB8, Porin and Neurofilament H or NDUFA13, SDHA and Neurofilament H) in PBS at 4 °C then a 4 day incubation with the biotin conjugated secondary antibodies (Anti-Mouse IgG, Fcγ Subclass 1 biotin conjugate or Anti-Mouse IgG, Fcγ Subclass 2b biotin conjugate) at 4 °C. Finally the fluorophores (Streptavidin, Alexa Fluor 546 conjugate, Alexa Fluor 647 anti-mouse IgG2b and Alexa Fluor 488 anti-rabbit IgG or Streptavidin or, Alexa Fluor 546 conjugate, Alexa Fluor 647 anti-mouse IgG1 and Alexa Fluor 488 anti-rabbit IgG) were applied for 4 days at 4 °C before processing for imaging.

### Imaging

In preparation for imaging, cerebellar sections were washed 5 × 30 minutes in PBS before a 30 minute incubation in 0.2 M glycine and an overnight incubation in Refractive Index Matching Solution (RIMS, 40 g Histodenze (Sigma Aldrich), 30 ml Phosphate Buffer (0.62 g Sodium dihydrogen phosphate 2.18 g Sodium hydrogen phosphate 1 L _d_H_2_O pH 7.4), 0.1% tween-20 (Sigma Aldrich)). For the purpose of imaging the sections were placed between two coverslips. Cerebellar sections were imaged (Z-stack volume, 250 μm) with an X20 objective (0.70 numerical aperture) on a confocal laser microscope (Nikon A1R, Nikon instruments) using the Galvano settings at excitation wavelengths of 488 nm, 546 nm and 647 nm. NIS Elements viewer software (Nikon instruments) was used to view the images post capture while Imaris software (Bitplane) was used for the 3D rendering modifications.

## Additional Information

**How to cite this article**: Phillips, J. *et al*. Development of passive CLARITY and immunofluorescent labelling of multiple proteins in human cerebellum: understanding mechanisms of neurodegeneration in mitochondrial disease. *Sci. Rep.* **6**, 26013; doi: 10.1038/srep26013 (2016).

## Supplementary Material

Supplementary Information

Supplementary Video 1

## Figures and Tables

**Table 1:** Details of the primary antibodies trialled on 250 μm mouse cerebellar sections and summary of the results.

Primary Antibody	Target	Host	Isotype	Dilution	Manufacturer and product code	Quality of Staining
anti-Neurofilament H 200 kDa	Neurofilament H (200 kDa)	Rabbit	IgG	1:200	Millipore (AB5539)	Specific labelling of axons
Anti-Myelin basic protein	Myelin basic protein	Mouse	IgG1	1:500	Biolegend (836502)	Specific labelling of myelin
anti-NDUFA13	NDUFA13	Mouse	IgG2b	1:100	Abcam (ab110240)	Specific positive staining of mitochondria
Anti-Porin	Porin	Mouse	IgG2b	1:100	Abcam (ab14734)	Specific positive staining of mitochondria
Anti-complex IV subunit I	COX1	Mouse	IgG2a	1:100	Abcam (ab14705)	Specific positive staining of mitochondria
anti-complex IV subunit IV	COX4	Mouse	IgG2a	1:100	Abcam (ab14744)	Specific positive staining of mitochondria
anti-SMI-31	Phosphorylated neurofilament H & M	Mouse	IgG1	1:1000	Biolegend (801601)	Specific labelling of axons
anti-SDHA	Succinate dehydrogenase complex, Subunit A (complex II)	Mouse	IgG1	1:100	Abcam (ab14715)	Specific positive staining of mitochondria
anti-parvalbumin	Parvalbumin	Mouse	IgG1	1:100	Swant (235)	Specific labelling of Purkinje Cell soma
anti-calbindin D-28k	Calbindin	Mouse	IgG1	1:100	Swant (235)	Specific labelling of Purkinje Cell soma
Anti-glut-1	Endothelial cells	Rabbit	IgG	1:100	Thermofisher (PA1–21401)	Staining of both arterioles and capillaries
Anti-α smooth muscle actin	Smooth muscle cells	Mouse	IgG2a	1:100	Dako (M8051)	In formalin-fixed sections, the smooth muscle layer in arterioles are clearly labelled but there is non-specific staining of nuclei. This non-specific staining is not observed in the 4% PFA-fixed and sucrose frozen section.

The details of primary antibodies that are commonly used in our laboratory and a summary of the results when applied 250 μm thick mouse cerebellar sections that have been passively cleared.

**Table 2:** Tissue details for the control subjects and patients with mitochondrial disease that are used in this study.

	Gender	Age (Years)	PMI (Hours)	Formalin-fixed/PFA-sucrose frozen	Fixation (years)	Genetic defect	Cause of Death	Publications
Control 1	F	72	27	Formalin-fixed	7	NA	Pulmonary oedema.	
Control 2	F	58	39	Formalin-fixed	8	NA	Cushing’s disease.	
Control 3	M	65	28	PFA-sucrose frozen	15 weeks	NA	Respiratory failure due to acute bronchial asthma.	
Patient 1	M	30	21	Formalin-fixed	6	m.3243 A > G	Left ventricular failure.	13,14
Patient 2	M	58	66	Formalin-fixed	3	m.8344 A > G	Stroke-like episode.	13,14
Patient 3	M	79	85	Formalin-fixed	4	*POLG* (p.Thr251Ile/p.Pro587Leu; p.Ala467Thr)	Pneumonia due to mitochondrial disease.	13,14
Patient 4	M	55	112	Formalin-fixed	3	*POLG* (p.Trp748Ser and p.Arg1096Cys)	Complications due to mitochondrial disease.	13,14
Patient 5	F	60	39	Formalin-fixed	4	Multiple mtDNA deletions due to an unconfirmed nDNA defect	Pulmonary embolus.	
Patient 6	M	47	76	Formalin-fixed	2	m.3243 A > G	Cardiac arrest.	

The details which include age, gender, Post mortem interval (PMI), fixation length and cause of death from tissue that was supplied by NBTR. NA – Not applicable.
