# Supplementary material for: Development of passive CLARITY and immunofluorescent labelling of multiple proteins in human cerebellum: understanding mechanisms of neurodegeneration in mitochondrial disease
Source: Sci Rep. 2016 May 16;6:26013. doi: 10.1038/srep26013 (PMC4867607; doi:10.1038/srep26013)
Supplement: Supplementary Information [file srep26013-s1.pdf]

Development of passive CLARITY and immunofluorescent labelling of multiple proteins in human cerebellum: understanding mechanisms of neurodegeneration in mitochondrial disease.

Jonathan Phillips[1], Alex Laude[2], Robert Lightowlers[1, 3], Chris M. Morris[4], Doug M. Turnbull[1], Nichola Z Lax[1*]

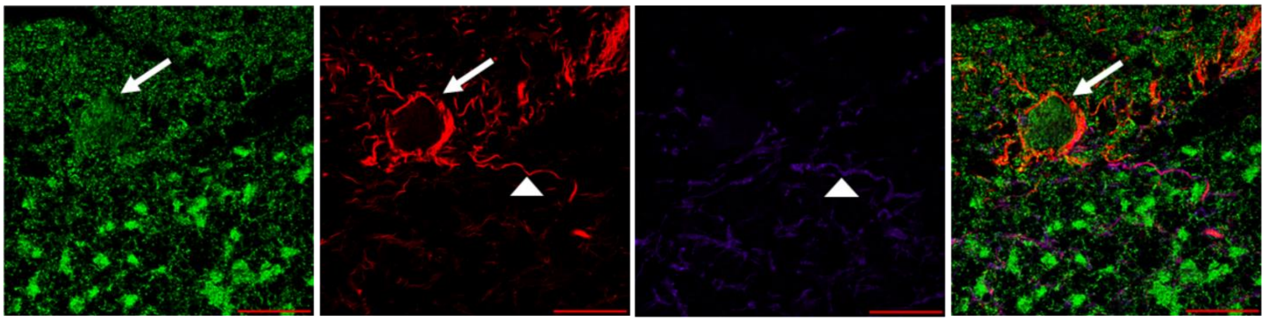

Supplementary Figure 1: Immunofluorescent staining of mitochondria in 5µm thick human cerebellar sections.

Mitochondria (porin; green) are clearly labelled in the Purkinje cell soma (arrow), while myelinated (MBP; purple) axons (NF-H; red) are observed in the granule cell layer (arrow head). Scale: 50µm.

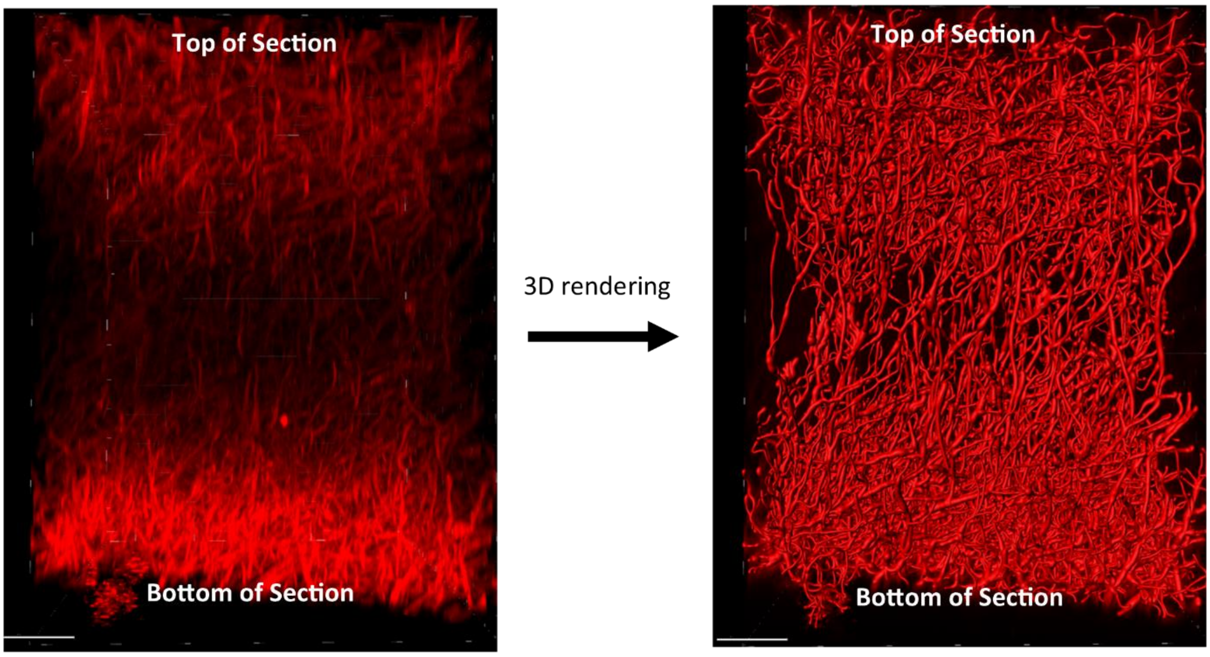

Supplementary Figure 2: Poor antibody penetration in 500µm thick in mouse cerebellum section.

*Poor antibody penetration is observed in 500µm-thick cerebellum with strong positive labelling of myelin basic protein (**a**; red; 546 nm) in the first and last 150µm of the section with reduced signal intensity in the middle of the tissue. This is further exemplified by 3D rendering of the image using the Imaris software, where the dense complex network of myelin can be observed at the surfaces/edges however in the middle there is a reduction in the density of myelin (**b**). Scale: 100µm.*

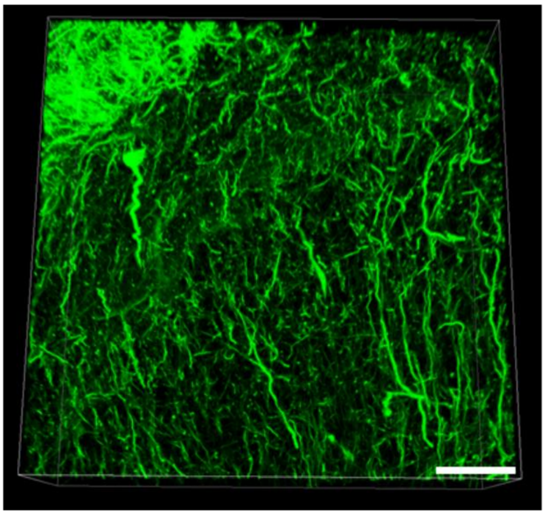

Supplementary Figure 3: Successful immunostaining of passively cleared 250µm thick human paraformaldehyde-fixed and cryoprotected cerebellar section.

Passive clearing and immunofluorescent labelling of tissue that underwent fixation PFA following by cryoprotection was tested in control 3. 250-µm-thick cerebellum tissue was stained for neurofilament H to identify Purkinje cells and their axons. Scale: 100µm.

Supplementary Video 1: Three-dimensional visualisation of the vascular network in passively cleared human cerebellum tissue.

A 250µm thick tissue of cerebellum of post-mortem human brain tissue was passively cleared and immunostained for endothelium (glut-1; green; 488 nm) and smooth muscle (α smooth muscle actin; red; 546 nm) to identify capillaries and arterioles respectively. Immunofluorescent labelling of this large volume of tissue reveals the three dimensional structure of the cerebellar vascular network. Scale: 100µm.

Supplementary Tables

Supplementary Table 1: Summary of the Primary antibody properties used in the study.

A summary of the different primary antibodies that have been used on passively cleared sections of both human and mouse cerebellar tissue.

Primary antibody	Target	Host	Isotype	Dilution	Manufacturer and product code
anti-Neurofilament H 200kDa	Neurofilament H (200 kDa)	Rabbit	IgG	1:200	Millipore (AB5539)
anti-NDUFA13	NDUFA13	Mouse	IgG2b	1:100	Abcam (ab110240)
anti-NDUFB8	NDUFB8	Mouse	IgG1	1:100	Abcam (ab110242)
anti-SMI-94	Myelin Basic Protein	Mouse	IgG1	1:500	Biolegend (836502)
anti-porin	porin	Mouse	IgG2b	1:100	Abcam (ab14734)
Anti-COX1	COX1	Mouse	IgG2a	1:100	Abcam (ab14705)
anti-COX4	COX4	Mouse	IgG2a	1:100	Abcam (ab110261)
anti-SMI-31	Phosphorylated neurofilament H & M chain	Mouse	IgG1	1:1000	Biolegend (801601)
anti-glut-1	Glut-1 (endothelial)	Rabbit	IgG	1:100	ThermoScientific (PA1-21041)
anti-SDHA	Succinate Dehydrogenase Complex, Subunit A	Mouse	IgG1	1:100	Abcam (ab14715)
anti-α smooth muscle actin	α smooth muscle actin	Mouse	IgG2a	1:100	Dako (M8051)
anti-parvalbumin	Parvalbumin	Mouse	IgG1	1:100	Swant (235)
anti-calbindin D-28k	Calbindin D-28k	Mouse	IgG1	1:100	Swant (300)

Supplementary Table 2: Description of secondary antibodies used.

A summary of the different secondary antibodies that have been used on passively cleared sections of both human and mouse cerebellar tissue.

Secondary antibody	Target	Host	Isotype	Dilution	Manufacturer
Alexa Fluor 633 anti-mouse IgG1	Mouse IgG1	Goat	IgG	1:100	Thermo Fisher Scientific
Alexa Fluor 633 anti-mouse IgG2a	Mouse IgG2a	Goat	IgG	1:100	
Alexa Fluor 647 anti-mouse IgG1	Mouse IgG1	Goat	IgG	1:100	
Alexa Fluor 647 anti-mouse IgG2b	Mouse IgG2b	Goat	IgG	1:100	
Alexa Fluor 405 anti-rabbit IgG	Rabbit IgG	Goat	IgG	1:100	
Alexa Fluor 488 anti-rabbit IgG	Rabbit IgG	Goat	IgG	1:100	
Alexa Fluor 488 anti-mouse IgG1	Mouse IgG1	Goat	IgG	1:100	
Alexa Fluor 488 anti-mouse IgG2b	Mouse IgG2b	Goat	IgG	1:100	
Alexa Fluor 488 anti-mouse IgG2a	Mouse IgG2a	Goat	IgG	1:100	
Alexa Fluor 546 anti-mouse IgG1	Mouse IgG1	Goat	IgG	1:100	
Alexa Fluor 546 anti-rabbit IgG	Mouse IgG1	Goat	IgG	1:100	
Streptavidin, Alexa Fluor 546 conjugate	Biotin	Goat	IgG	1:100	
Anti-Mouse IgG, Fcγ Subclass 1 biotin conjugate	Mouse IgG1	Goat	IgG	1:100	Jackson Laboratories
Anti-Mouse IgG, Fcγ Subclass 2b biotin conjugate	Mouse IgG2b	Goat	IgG	1:100	Jackson Laboratories
